# Intestinal Epithelial Cell Endoplasmic Reticulum Stress and Inflammatory Bowel Disease Pathogenesis: An Update Review

**DOI:** 10.3389/fimmu.2017.01271

**Published:** 2017-10-25

**Authors:** Xiaoshi Ma, Zhaolai Dai, Kaiji Sun, Yunchang Zhang, Jingqing Chen, Ying Yang, Patrick Tso, Guoyao Wu, Zhenlong Wu

**Affiliations:** ^1^State Key Laboratory of Animal Nutrition, Department of Animal Nutrition and Feed Science, China Agricultural University, Beijing, China; ^2^Department of Pathology and Laboratory Medicine, Metabolic Diseases Institute, University of Cincinnati, Cincinnati, OH, United States; ^3^Department of Animal Science, Texas A&M University, College Station, TX, United States; ^4^Beijing Advanced Innovation Center for Food Nutrition and Human Health, China Agricultural University, Beijing, China

**Keywords:** intestinal epithelial cells, unfolded protein response, endoplasmic reticulum stress, immune response, intestinal bowel disease, colitis

## Abstract

The intestinal epithelial cells serve essential roles in maintaining intestinal homeostasis, which relies on appropriate endoplasmic reticulum (ER) function for proper protein folding, modification, and secretion. Exogenous or endogenous risk factors with an ability to disturb the ER function can impair the intestinal barrier function and activate inflammatory responses in the host. The last decade has witnessed considerable progress in the understanding of the functional role of ER stress and unfolded protein response (UPR) in the gut homeostasis and its significant contribution to the pathogenesis of inflammatory bowel disease (IBD). Herein, we review recent evidence supporting the viewpoint that deregulation of ER stress and UPR signaling in the intestinal epithelium, including the absorptive cells, Paneth cells, goblet cells, and enteroendocrine cells, mediates the action of genetic or environmental factors driving colitis in experimental animals and IBD patients. In addition, we highlight pharmacologic application of chaperones or small molecules that enhance protein folding and modification capacity or improve the function of the ER. These molecules represent potential therapeutic strategies in the prevention or treatment of IBD through restoring ER homeostasis in intestinal epithelial cells.

## Introduction

As the largest barrier that separates the mammalian host from the external environment, gastrointestinal epithelia, including Paneth cells, goblet cells, enteroendocrine cells, and absorptive enterocytes, are critical factors that influence the intestinal homeostasis (1). Specifically, Paneth cells produce and secrete various antimicrobial peptides, which in turn regulate the composition of the intestinal microbiota and the ability to withstand intestinal pathogens (2, 3). Goblet cells are responsible for production of mucins, the predominant component of the intestinal mucus layer that prevents direct contact of luminal contents with epithelial cells (2, 4). The main function of enteroendocrine cells is to produce and secrete peptide hormones that modulate the motility of the digestive tract and metabolism. The absorptive epithelial cells are mainly associated with the secretion of a large number of cytokines and chemokines, which can regulate the composition of the commensal microbiota and the host immune responses (5). Intestinal homeostasis is primarily determined by the appropriate function of the intestinal epithelial cells. Consistently, dysfunction of intestinal epithelium is associated with the development of various gastrointestinal disorders, such as irritable bower syndrome, inflammatory bowel disease (IBD), celiac disease, and mucosal disease (1, 5, 6).

Inflammatory bowel disease, a chronic inflammatory disorder that is mainly composed of Crohn’s disease (CD) and ulcerative colitis (UC), is characterized by abdominal pain, diarrhea, and bloody stools (7–9). Despite well-defined clinical manifestations, the etiology of IBD remains largely unknown. It is generally believed that IBD is a multifactorial gastrointestinal disorder in which various factors, such as genetic factors, intestinal microbiota, host immune responses, and environmental factors are involved (8, 10). Recent studies have shown that endoplasmic reticulum (ER) stress and the unfolded protein response (UPR) are critical factors associated with susceptibility to IBD and intestinal homeostasis (11, 12). The effect of ER stress on the pathogenesis of IBD is majorly mediated by impairing the mucosal barrier function, regulating innate or adaptive immune response of the host cells, and modulating the intestinal microbiota (13, 14). These findings link ER and IBD, therefore advancing our understanding of IBD pathogenesis and proposing novel therapeutic strategies by restoring ER function in intestinal epithelial cells. Herein, we will review the functional roles of ER in the intestinal homeostasis, and how this homeostasis is impaired by genetic or environmental factors and contributes to susceptibility to IBD. Potential therapeutic interventions targeting ER stress signaling are also reviewed.

## The ER and UPR Signaling

The ER is the major site for the synthesis and folding of membrane and secretory proteins (13, 15). In addition, the ER is associated with lipid biosynthesis, energy metabolism, and homeostasis of intracellular Ca^2+^ (12). Impairment of the ER function causes a cellular condition known as ER stress (16). Mammalian cells have evolved a series of signal transduction pathways to eliminate the deleterious effects, which are collectively termed as the UPR. Activation of UPR is an adaptive response for mammalian animals to restore ER homeostasis and survive the stressful conditions by blocking global mRNA translation, eliminating misfolded proteins by ER-associated protein degradation (ERAD) signaling pathway, and enhancing the capacity for protein folding and modification (16, 17). However, severe or prolonged ER stress can activate cell death signaling to remove damaged cells (12, 18, 19).

**Figure 1:**
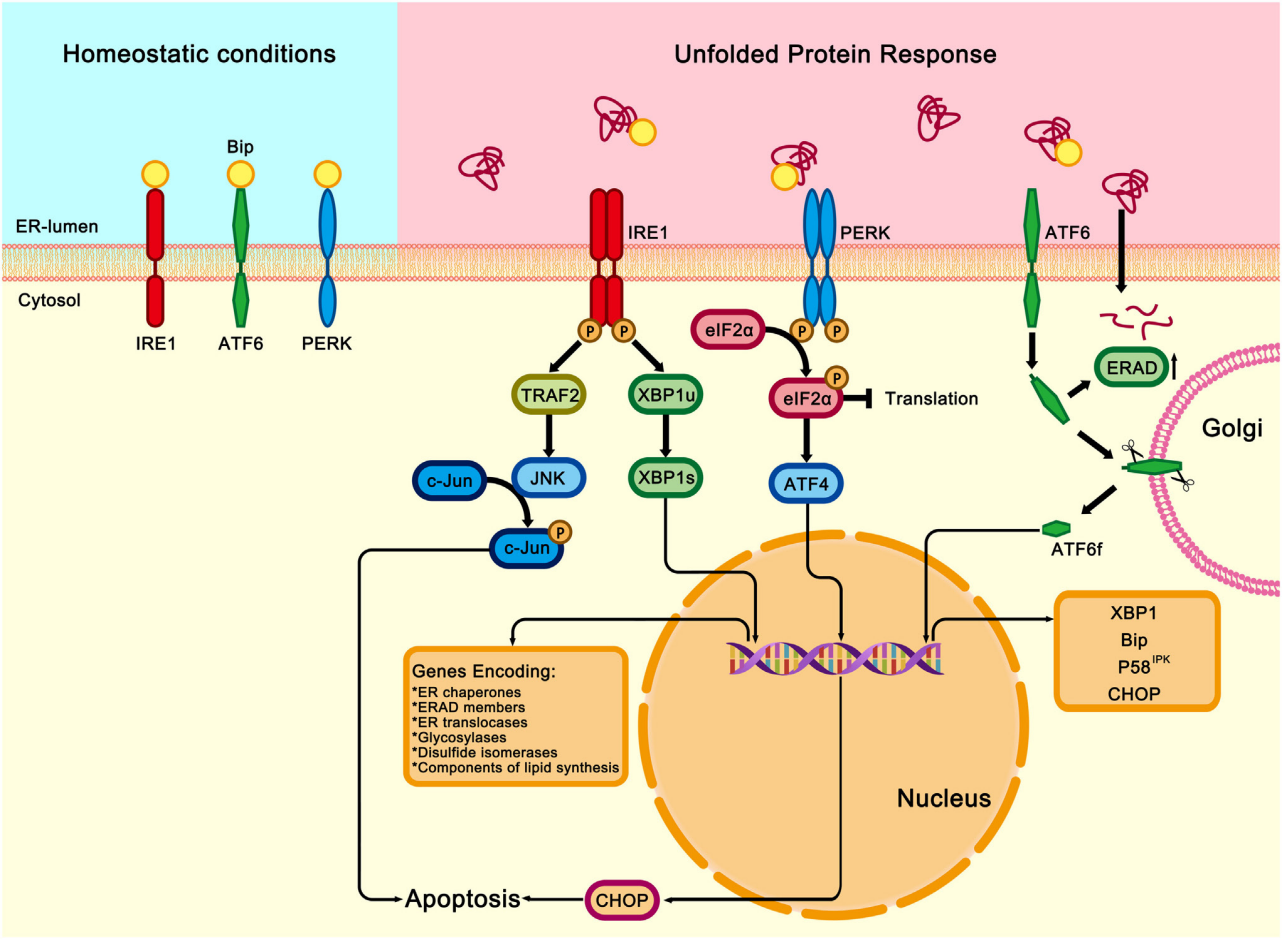
Three signaling pathways of unfolded protein response (UPR). Under homeostatic conditions, immunoglobulin heavy chain-binding protein (Bip) binds and inhibits the three transmembrane proteins of UPR: the inositol-requiring transmembrane kinase/endonuclease 1 (IRE1), the pancreatic endoplasmic reticulum (ER) kinase-like ER kinase (PERK), and activating transcription factor 6 (ATF6). Under ER stress conditions, Bip dissociates from the three transmembrane proteins and binds to the misfolded or unfolded proteins in the ER, which activates IRE1, PERK, and ATF6 downstream signalings. Once released from Bip, IRE1 is activated through homodimerization and trans-autophosphorylation. The activated IRE1 slices the X-box binding protein (XBP1u) and generates a functionally active isoform of XBP1 (XBP1s). XBP1s is a transcription factor that modulates the expression of genes encoding ER chaperones, ER-associated protein degradation (ERAD) members, ER translocases, glycosylases, disulfide isomerases, and components involved in lipid biosynthesis. IRE1 also binds to and activates TNFα receptor associated factor 2 (TRAF2), which results in activations of c-Jun N-terminal kinase (JNK), therefore contributing to inflammatory, proapoptotic signaling in response to the ER stress. PERK is also activated by homodimerization and trans-autophosphorylation. Activated PERK phosphorylates the eukaryotic translation initiation factor 2 (eIF2α), thereby attenuating global protein synthesis and alleviating the burden on ER. However, the transcription factor ATF4 can bypass the inhibition and activate the expression of *Chop*, which is a master regulator of ER stress-induced apoptosis. After disassociation from ATF6. Bip moves to the Golgi apparatus, where it subsequently undergoes intramembrane proteolysis in its luminal domain. The released ATF6 fragment (ATF6f) translocates to the nucleus and regulates the expression *XBP1, Bip, P58^IPK^*, and *Chop*.

In eukaryotic cells, UPR signaling pathways are mainly mediated by three protein sensors on the ER membrane: inositol-requiring transmembrane kinase/endonuclease 1 (IRE1), pancreatic ER eIF2α kinase (PERK), and activating transcription factor 6 (ATF6) (20–22) (Figure 1). Under non-stressed conditions, all three transmembrane sensors are bound to the ER chaperone binding immunoglobulin protein (Bip, also known as glucose-regulated protein 78) in their intraluminal domains and are maintained in an inactive state (23–25). Upon ER stress, Bip dissociates from the luminal domains of the three protein sensors, therefore activating IRE1, PERK, or ATF6, and initiating UPR and downstream cascade signaling (26, 27).

### IRE1 Signaling

Among the three protein sensors, IRE1 is the most evolutionarily conserved ER stress transducer protein (20, 28). IRE1 exists in two structurally related isoforms, IRE1α, the ubiquitously expressed isoform, and IRE1β, which has been primarily identified in the intestinal epithelium of the gut and respiratory tract (29, 30). Upon sensing the misfolded or unfolded proteins, Bip protein dissociates from IRE1α and facilitates the activation of IRE1α through homodimerization and trans-autophosphorylation or other mechanisms (31, 32). Activated IRE1α uses its endoribonuclease activity to remove a 26-bp pair segment from an unspliced mRNA encoding the transcription factor X-box binding protein 1 (XBP1u), which in turn causes a shift in the reading frame and generates a spliced and functionally active isoform of XBP1 (XBP1s) (33, 34). XBP1s is a potent CREB/ATF basic leucine zipper (bZIP) transcription factor that can induce the expression of genes involved in protein folding, secretion, maturation, the ERAD signaling and the synthesis of phospholipids (26, 35). Several lines of studies have shown that XBP1s is also implicated in various biological processes, such as lipid metabolism (36), pro-inflammatory cytokines synthesis (37), the hypoxia response signaling pathway (38), cellular differentiation (39), and the hexosamine biosynthetic pathway (40), indicating a critical role for IRE1-XBP1 UPR signaling and cellular response. Unlike XBP1s, XBP1u is a short-lived protein that lacks the transactivation domain. XBP1u inhibits the translocation of XBP1s from cytoplasm into the nucleus, therefore serving as a dominant-negative regulator to block the transactivation of XBP1 downstream targets under certain conditions (41–43). Recent studies have shown that IRE1α can target other mRNAs for degradation, therefore inhibiting the synthesis of nascent proteins through the regulated IRE1-dependent decay (19, 44, 45), indicating an additional level of regulation to cope with ER stress.

It should be noted that the functional role of IRE1α on various biological processes, such as proliferation, metabolism, inflammation, autophagy, and apoptosis, can be mediated in an XBP1-independent manner (46). First, IRE1α can bind to and activate TNFα receptor associated factor 2 in the cytoplasm, which in turn activates c-Jun N-terminal kinase or nuclear factor-κB (NF-κB), thus participating in inflammatory response or proapoptotic signaling in response to ER stress (12, 17, 18). Second, IRE1α can directly modulate p38 MAPK and ERK1/2, two critical protein kinases related to stress response, indicating a link between UPR signaling and cellular response (47). Third, IRE1α has been reported to interact with the proapoptotic BCL-2 (B-cell lymphoma 2) family proteins, BAX (BCL-2-associated X protein), or BAK (BCL-2 antagonist/killer), therefore contributing to apoptotic cell death (48). In addition to transcription regulation and protein interactions, IRE1α can cause degradation of selective microRNA that normally represses translation of caspase-2, therefore leading to activation of the mitochondrial apoptosis pathway (49–51). More studies are required to elucidate how this epigenetic regulation is implicated in and contribute to intestinal homeostasis. In contrast to IRE1α, IRE1β has a broader endoribonuclease activity, which can lead to the degradation of a large array of transcripts (52). However, the underlying mechanisms are incompletely understood.

### PERK Signaling

PERK has structural similarities with IRE1 transmembrane protein (21, 53). Disengagement from Bip upon sensing misfolded or unfolded protein in the ER activates PERK by homodimerization and trans-autophosphorylation (11, 54, 55). Activated PERK phosphorylates the α subunit of eukaryotic translation initiation factor 2 (eIF2α), a component of the translation initiation complexes, therefore attenuating global protein synthesis and alleviating the overload of misfolded proteins (53, 56). Interestingly, the phosphorylated eIF2α selectively enhances translation of ATF4, a transcription factor regulating the expression of genes implicated in protein folding, oxidative stress response, and ER stress-induced apoptosis (57, 58). CHOP (CCAAT/enhancer-binding protein homologous protein, also known as GADD153) has been shown to be a critical mediator responsible for ER stress-induced cell death through different mechanisms (19, 59). First, CHOP can suppress the antiapoptotic protein BCL-2 or enhance numerous proapoptotic proteins, such as Bim, telomere repeat binding factor 3, GADD34 (growth arrest and DNA damage 34), or death receptor 5 (60–62). Second, CHOP induces the transcriptional expression of ER oxidase 1α, which in turn resulted in generation of reactive oxygen species (ROS) and release of Ca^2+^ from the ER, thereby conferring to apoptosis (12, 18, 19). Third, CHOP can interact with ATF4 and activate genes involved in protein synthesis machinery, thus causing energy depletion and apoptosis in ER-stressed cells (63). In addition to inducing cell death, activation of the PERK pathway can activate antioxidant reactions to avoid accumulation of ROS in response to ER stress (64). This effect of PERK is mediated by ATF4-induced phosphorylation of nuclear factor-erythroid-derived 2-related factor 2, which can activate enzymes with an ability to remove oxidants. These enzymes include NAD(P)H-quinone oxidoreductase, heme oxygenase 1, and glutathione *S*-transferase (65, 66).

Besides PERK, eIF2α can be phosphorylated by other protein kinases, including PKR (double-stranded RNA activated protein kinase) (67), general control non-depressive kinase 2 (68), and heme-regulated inhibitor kinase (69). All the protein kinases have similar kinase catalytic domains, and therefore possess a capability to phosphorylate eIF2α at its Ser 51 residue to regulate protein synthesis (70). Because of the presence of different regulatory domains in the kinases, they can be activated by different stress stimuli (70). Despite the diversity of stress stimuli and activated protein kinases, these signaling cascades converge on the phosphorylation of eIF2α, indicating a critical functional role of eIF2α in determining cell fate decision. These biochemical roles of eIF2α have been highlighted in several review papers (70, 71). Additional studies are needed to elucidate how the kinases-activate eIF2α interacts with ER stress signaling and contribute to cell survival and apoptosis under specific conditions.

Importantly, eIF2α can be dephosphorylated by protein phosphatase, such as protein phosphatase 1 regulatory subunit 15A (growth-arrest DNA damage-inducible protein 34, also known as GADD34) and subunit 15B (known as CReP) (72–74), thus forming a negative feedback regulation on PERK–eIF2α signaling. All these data indicate that activation of eIF2α acts as a molecular switch either to induce cell death or to promote cell survival by attenuating protein synthesis in a context-dependent manner (63).

### ATF6 Signaling

Activating transcription factor 6 is a key transcription factor that helps intestinal epithelial cells cope with ER stress (22). Two homologous ATF6 proteins, such as ATF6α and ATF6β, have been identified in mammalian cells (75). Upon sensing the misfolded or unfolded proteins in the ER, ATF6 that is released from Bip migrates from the ER to the Golgi apparatus, where it subsequently undergoes cleavage by site-1 protease (S1P) and site-2 protease (S2P) in its luminal domain and transmembrane region, respectively, leading to the release of the cytosolic domain of ATF6, ATF6 fragment (ATF6f) (22, 24). ATF6f then translocates to the nucleus to bind DNA and transcriptionally upregulates target genes involved in protein folding, or ERAD to restore ER homeostasis or induce cell death in response to severe or prolonged ER stress (76–79).

In addition to the canonical ER membrane-bound proteins, IRE1, PERK, and ATF6 as abovementioned, novel types of ER stress transducers sharing a region of high sequence similarity with ATF6 have been identified (80, 81). These proteins possess a transmembrane domain, which allows them to associate with the ER, and have a transactivation domain and a basic leucine zipper (bZIP) domain. They are collectively known as old astrocyte specifically induced substance (OASIS) family members, which consist of CREB3L1/OASIS (82), CREB3L4/CREBH (RE-Bip H) (83), CREB3L2/BBF2H7 (box B-binding factor 2 human homolog on chromosome 7) (84), AIbZIP/Tisp40/CREB3L4/CREB4 (cyclic AMP responsive element Bip 4) (85), and Luman/LZIP/CREB3 (86, 87). Most of these ATF6-related bZip factors are processed at the Golgi as described for ATF6, but their functions are tissue specific due to the unique cell or tissue specific expression patterns of these transducers (88, 89).

## Intestinal Epithelial cell and ER Stress in IBD

The gut epithelial cells are constantly exposed to a complex microenvironment involving intestinal microbiota, antigens, dietary metabolites, and bacterial toxins (90). Among the epithelial cells, enterocytes are the major cell types that are replaced in a short period, which require a high metabolic rate and biosynthesis of large amounts of proteins, cytokines, and small peptides. As the major secretory cells, goblet cells and Paneth cells can produce and secret mucin glycoproteins which are the major components of mucus that separate the luminal microbial flora from the intestinal epithelium and lubricates the epithelium (4, 91). They can also secrete defensins, lysozymes, antimicrobial lectins, collectins, and smaller amounts of MUC2 (2, 92). Under physiological conditions, the secretion of antimicrobial peptides and mucins with large numbers of disulfide bonds and/or homo-oligomerization can be maintained in homeostasis due to appropriate ER function in intestinal epithelium (90, 92). In response to environmental factors, such as pathogenic bacteria infection, the production of MUC2 or defensins can be stimulated in the secretory cells (93, 94), thus exerting a significant protein folding and modification burden on ER in IECs. This burden and the complexity of the intestinal environment may pose particular challenges to the capacity of proteins for folding in intestinal epithelial cells and results in ER stress and activation of UPR survival signaling or induction of cell death if the ER homeostasis could not restored (95). In addition, ER stress in intestinal epithelial cells is associated with activation of host immune response and intestinal dysbiosis, which are critical factors implicated in the pathogenesis of intestinal diseases including IBD and mucosal disease (14, 90, 96). Importantly, a genetic deficiency of genes involved in UPR results in higher susceptibility to IBD due to decreased capacity to reduce the concentrations of unfolded proteins in the ER, as well as overactivated immune response in epithelial cells (55, 97–99) (Table 1).

**Table 1:** Role of endoplasmic reticulum stress and secretion-related genes in inflammatory bowel disease.

Gene	Disease	Possible mechanism	Reference
*IRE1*α	Spontaneous colitis	Increased CHOP-related apoptosis	(103)
*Xbp1*	Spontaneous enteritis	Increased CHOP-related apoptosis	(99)
*P58^IPK^*	Dextran sodium sulfate (DSS)-induced colitis	Increased CHOP-related apoptosis	(11)
*Atf6*α	DSS-induced colitis	Decreased binding protein (Bip) expression	(11)
*Mbtps1*	DSS-induced colitis	Decreased Bip and Grp94 expression	(123)
*Muc2*	Spontaneous colitis	Nuclear factor-κB and apoptosis activation	(127)
*Agr2*	Severe ileitis and colitis	Increased CHOP-related apoptosis	(55)

### UPR Regulators and IBD Pathogenesis

#### IRE1/XBP1 Signaling in IBD

Initial evidence linking IRE1/XBP1 signaling to intestinal inflammation came from a study showing that genetic deletion of *IRE1*β increased the protein level of Bip in the colonic mucosa and susceptibility to dextran sodium sulfate (DSS), a well-known inducer of experimental colitis in mice (30). Further study has shown that *IRE1*β knockout mice exhibit impaired intestinal barrier function and aberrant accumulation of mucin due to the deficiency of the negative feedback control on mucin by IRE1β in goblet cells (100). Similarly, genetic deletion of IRE1α in IECs leads to spontaneous colitis, which is accompanied by loss of goblet cells and dysregulated epithelial barrier function (101). Moreover, *IRE1*α*^−/−^* mice are more susceptible to DSS-induced colitis and ER stress-related apoptosis (101). XBP1 is a critical effector transcription factor of IRE1 signaling in response to ER stress and unfolded protein accumulation. It is not a surprise that the *XBP1* gene on chromosome 22q12 has been linked to IBD for more than two decades (102, 103). The deep sequencing of *Xbp1* and its promoter revealed more single nucleotide polymorphisms (SNPs) in both UC and CD patients than in healthy controls (97). These SNPs in *Xbp1* were found to be associated with decreased transactivation of XBP1-regulated UPR target genes and increased inflammatory response. The functional role for XBP1 in IBD was further validated in *Xbp1^−/−(IEC)^* (genetic depletion of *Xbp1* in the epithelium of the small and large intestines) mice, as evidenced by spontaneous development of intestinal inflammation and increased sensitivity to DSS (97). Moreover, *Xbp1^−/−(IEC)^* mice have leaky intestinal barrier, increased translocation of invading pathogens to the liver and other tissues, indicating an essential role of Xbp1 in the intestinal homeostasis and host immune response, which might act in concert and contribute to IBD (97).

#### PERK/CHOP Signaling in IBD

CHOP is a transcription factor implicated in both apoptosis and inflammatory responses (104). Elevated expression of CHOP has been observed in the intestinal epithelium of IBD patients and mice with deficiency in *Xbp1, Atf6*α, or *P58^IPK^* (11, 97). Park et al. reported that ER stress-activated CHOP can suppress peroxisome proliferator-activated receptor γ, a negative regulator of NF-κB, therefore resulting in NF-κB activation (105). Activated NF-κB translocates into the nucleus and enhanced the production of interleukin-8, a pro-inflammatory cytokine in intestinal epithelium, which in turn contributes to intestinal dysfunction and IBD (105). In addition to a regulatory effect on cytokines, CHOP can promote the infiltration of macrophages, induce ROS and IL-1β production, or enhance apoptosis of epithelial cells, thus leading to the development of colitis (106).

In addition to IL-8 and IL-1β, interleukin 23 (IL-23) has been identified as a critical cytokine in the pathogenesis of IBD (107). Polymorphisms in IL-23 receptor (*IL-23R*) have been reported in both CD and UC patients (108). Further study shows that IBD-affected individuals have an increased concentration of IL-23 in the inflamed epithelium, indicating a potential role for IL-23 in intestinal immune response (109). The pro-inflammatory activity of IL-23 has mostly been linked to its effect on Th17 cells, a population of T cells characterized by the production of the inflammatory cytokine interleukin 17 (IL-17) (110). The functional role for the IL-23/IL-17 axis in intestinal inflammation has been validated in various animal models (110, 111). In addition, IL-23 can also regulate the activity of regulatory T cells, therefore modulating the host immune system (112–116). In a recent study, the CHOP protein has been reported to enhance TLR-induced IL-23 production by enhancing the binding to the IL-23 p19 (*Il23A*) promoter in ER-stressed myeloid cells (117). Further study is needed to explore whether this transcriptional regulation is implicated in and contributes to intestinal inflammation.

#### ATF6 Signaling in IBD

An appropriate function of ATF6α is required to survive chemical-induced ER stress in mice (118). This effect of ATF6α is mediated by transcriptional activation of ER chaperone genes, including *Bip, Grp94*, and *P58^IPK^* (64, 119, 120). ATF6α knockout (*Atf6*α*^−/−^*) mice display reduced expression of ER chaperone genes, and increased expression of a proapoptotic protein CHOP in colonic epithelium (11). *P58^IPK^* knockout mice have a decreased number of goblet cells, increased inflammatory cell infiltration, and more severe mucosal damage upon DSS challenge (11). These data highlight the requirement of ATF6 signaling for intestinal barrier function and inflammatory response.

The functional role of ATF6 signaling pathway in the pathogenesis of IBD has been revealed by the using of mice with mutations in *Mbtps1*, a gene encoding ATF6 activator S1P (121). These mice exhibited decreased protein levels of Bip, Grp94, and impaired ATF6-driven UPR to DSS administration (121). It should be noted that S1P can also activate OASIS, a bZIP transcription factor implicated in colitis by interfering with ER stress signaling (122). Moreover, *Oasis^−/−^* mice have been reported to have similar phenotypes to those observed in ATF6 knockout mice, including impaired goblet cell function (123), and elevated apoptotic proteins in IECs (124). It is currently unknown which signaling pathway is a major contributor to the development of chemical-induced colitis in Mbtps1 deficiency mice. Additional studies with genetically engineered mice are needed to answer this question and a potential functional overlap between ATF6 and OASIS signaling.

### Protein Secretion-Related Factors in IBD

Paneth cells and goblet cells in the gastrointestinal tract can produce large amounts of proteins, which undergo protein folding and posttranslational modifications before being secreted from the cells (90, 92). This feature of secretory cells requires a fine monitoring and management of the ER to avoid the accumulation of unfolded or misfolded proteins. Both clinical and experimental animal studies show that impaired UPR is associated with development of colitis in humans and animals as abovementioned (11, 100, 123). Several lines of studies show that dysfunction of genes involved in protein secretion, such as *Anterior gradient 2 (Agr2)* and MUC2, is associated with IBD through various mechanisms (98, 125).

*Agr2* is an ER-resident protein expressed in secretory IECs such as goblet, Paneth, and enteroendocrine cells in the small intestine (126). This protein responsible for the formation of correctly arranged disulfide bonds in mature proteins (127, 128). By using inducible *Agr2^−/−^* mice, Fang et al. showed that deletion of *Agr2* is associated with decreased goblet cells and MUC2, dramatic expansion of the Paneth cell compartment, abnormal Paneth cell localization, elevated ER stress, and severe colitis (125). This finding, along with previous observation that both CD and UC patients have decreased AGR2 (129), indicates that AGR2 is essential for intestinal homeostasis. Deficiency of this gene impairs the secretion of proteins in intestinal epithelial cells and activates uncontrolled immune response, ultimately contributing to IBD.

MUC2 is the major component of mucin that goblet cells secrete into the intestinal lumen (130, 131). An appropriate secretion of MUC2 requires extensive *O*-glycosylation of central mucin repeats and intra- and inter-chain disulfide bond formation in the cysteine-rich N and C-terminal domains within the ER (132). Mutations in the *Muc2* gene lead to ER stress, inflammation, and spontaneously colitis due to accumulation of MUC2 precursor in the ER and reduction in mucin secretion (98). Similar observations have been described in UC patients (131, 133). Interestingly, administration of interleukin-10 (IL-10), an anti-inflammatory cytokine, has been reported to attenuate intestinal inflammation and enhance mucin production in ER-stressed epithelial cells through currently unknown mechanisms (99).

### Environmental Factors and ER Stress Signaling in IBD

Besides the genetic factors and secretion-related proteins, various environmental factors have been implicated in ER stress signaling and contribute to intestinal inflammation in IBD (99, 134–136). Several inflammatory mediators have been shown to be able to influence ER stress response. For example, anti-inflammatory cytokine IL-10 can modulate ATF6 nuclear recruitment to the *Bip* promoter, therefore blocking ER stress (99). This finding provides a plausible explanation for the development of colitis in IL-10 knockout mice (137). By contrast, pro-inflammatory cytokine tumor necrosis factor α (TNF-α) can enhance ER stress and UPR signaling by inducing ROS production and its accumulation in the ER (134). Moreover, increased expression of inflammatory cytokines, such as IL-1β, TNF-α, and IFN-γ, has been observed in aberrant mucin assembly induced colitis mice (98). These findings highlight a role of immune response in the development of experimental colitis or IBD. More studies are required to uncover how the antagonistic cytokines are finely coordinated under physiological conditions and ER-stressed conditions.

Intestinal microbiota is considered to be another environmental factor highly correlated with IBD (138, 139). Intestinal microbiota is well known to produce inflammatory molecules by regulating expression of genes involved in immune response, which in turn triggers ER stress (55). In addition, the effect of microbiota on ER stress can also be mediated by various microbial metabolites. For example, trierixin, a macrocyclic lactam derived from *Streptomyces* sp., has been identified as an inhibitor of XBP1 splicing (135). Interestingly, two structurally related compounds, such as mycotrienin II and trienomycin A, were isolated from the culture broth of a trierixin-producing strain and reported to inhibit the induction of XBP1 (135). These compounds might be potential pharmacological tools for the functional analysis of XBP1 signaling in response to ER stress. However, key enzymes involved in the production of these metabolites with an ability to suppress XBP1, as well as their effects on the intestinal homeostasis, are still largely unknown. Paton et al. reported that oral infection with AB_5_ a cytotoxin-producing *Escherichia coli* resulted in pathologic UPR in mice (136). Further studies showed that the A subunit of AB_5_ cytotoxin specifically cleaved Bip by the serine protease activity and activated it in eukaryotic cells (140). Notably, proteins with significant sequence homology to the A and B subunits of this cytotoxin have been reported in a wide variety of microorganisms (136), which can interfere with the ER stress-related signaling cascade and inflammatory responses in the IECs.

## ER Stress and UPR in IBD Therapeutics

Therapies for IBD are faced with extraordinary challenges due to limited understanding of its etiology and pathogenesis (141). Experimental and clinical data have shown that deregulated ER stress signaling in intestinal epithelial cells is associated with development of UC or CD. In this scenario, chemical drugs or small molecules with the ability to reduce unfolded proteins or enhance the capacity of ER for protein folding and modification might be potential therapeutic strategies to prevent or treat IBD. Tauroursodeoxycholic acid (TUDCA), a bile acid derivative, and 4-phenylburyrate have been reported to enhance protein folding, ameliorate ER stress, and enhance insulin sensitivity in liver and muscle of obese patients (142–145). In a recent study, Cao et al. showed that oral administration of these two compounds dramatically alleviated DSS-induced inflammation and colitis *via* abolishing ER stress signaling in colonic IECs (11). A structure–function analysis revealed that ursodeoxycholic acid, the unconjugated form of TUDCA, is 10 times more effective in alleviating ER stress than TUDCA in IECs (146), and might be a potential drug to alleviate ER stress related colitis. It should be noted that this result is based on cell free assay; *in vivo* studies involving animals and clinical patients are required to validate this preliminary result. In another study, vaticanol B, a resveratrol tetramer, has been shown to suppress the induction of Bip, CHOP, and the secretion of TNF-α, indicating a beneficial effect by improving the ER function and maintaining the membrane integrity of the ER (147). In addition, fexofenadine, an antihistamine agent for allergic rhinitis and urticaria (148), has been found to prevent DSS-induced colitis by blocking ER stress-induced eIF2α and inhibiting NF-κB signaling in IECs through a histone receptor-independent signaling (149). Salubrinal, a specific inhibitor of eIF2α dephosphorylation, was found to ameliorate experimental colitis by boosting adaptive UPR signaling Bip, ATF4, and heat-shock protein 70 (150, 151). In addition to the chemical chaperones, nutritional interventions have gained increasing attention due to their regulatory effects on expression of genes implicated in ER stress. Glutamine, an abundant amino acid in tissues, has been reported to ameliorate 2,4,6-trinitrobenzenesulfonic acid (TNBS)-induced colitis by abrogating ER stress, reducing oxidative injury, attenuating apoptosis and the inflammatory response in colonic epithelial cell, and highlighting a potential nutritional strategy to restore ER function and improve intestinal homeostasis (152). In our recent study, we found that glutamine can regulate tight junction protein permeability through calcium/calmodulin-dependent kinase kinase 2 signaling in intestinal porcine epithelial cells (153). These findings suggest that supplementation with glutamine might be a promising adjuvant in IBD therapeutics. Considering that most of these data are results from *in vitro* or animal models. Further studies for the underlying mechanisms and clinical efficacy are warranted before these molecules can be used as a novel therapeutic option in IBD patients.

## Conclusion

Endoplasmic reticulum stress and related UPR signaling are implicated in and contribute to the initiation or progression of IBD. The last decade has witnessed considerable progress in the understanding of ER stress and UPR signaling in maintaining intestinal homeostasis. Dysfunction of the ER triggered by various factors is associated with susceptibility to of CD and UC and impairment in intestinal barrier. Reestablishing intestinal homeostasis by correcting ER stress-related signaling network is emerging as a potential therapeutic target for IBD. Considering that protein folding, posttranslational modification and trafficking are fundamental biological processes implicated in various physiological and pathological processes, manipulation of ER stress signaling without causing severe side effects is a challenge that must be carefully considered before its recommendation for the treatment of IBD patients. IBD is a chronic and relapsing inflammatory condition of the gastrointestinal tract, in which genetic factors, environmental factors, as well as an interplay between intestinal microbiota and the host immune response, contribute to the pathogenesis of the disease. An ideal therapy for IBD that is tailored to an individual’s specific condition highly depends on a deep understanding of phenotype, natural history, and the pathogenesis of this disease.

## Author Contributions

GW and ZW designed the study; XM, YZ, YY, and JC contributed to the literature search; XM, KS, and ZD drafted the manuscript; ZW, PT, and GW revised and finalized the manuscript.

## Conflict of Interest Statement

The authors declare that the research was conducted in the absence of any commercial or financial relationships that could be construed as a potential conflict of interest.

## References

[1] Pott J Hornef M. Innate immune signalling at the intestinal epithelium in homeostasis and disease. EMBO Rep (2012) 13(8):684–98. 10.1038/embor.2012.96 22801555 PMC3410395

[2] Ouellette AJ. Paneth cells and innate mucosal immunity. Curr Opin Gastroenterol (2010) 26(6):547–53. 10.1097/MOG.0b013e32833dccde 20693892

[3] Porter EM Bevins CL Ghosh D Ganz T The multifaceted Paneth cell. Cell Mol Life Sci (2002) 59(1):156–70. 10.1007/s00018-002-8412-z 11846026 PMC11337504

[4] Mcguckin MA Lindén SK Sutton P Florin TH. Mucin dynamics and enteric pathogens. Nat Rev Microbiol (2011) 9(4):265–78. 10.1038/nrmicro2538 21407243

[5] Catalioto RM Maggi CA Giuliani S. Intestinal epithelial barrier dysfunction in disease and possible therapeutical interventions. Curr Med Chem (2011) 18(3):398–426. 10.2174/092986711794839179 21143118

[6] Okumura R Takeda K. Roles of intestinal epithelial cells in the maintenance of gut homeostasis. Exp Mol Med (2017) 49(5):e338. 10.1038/emm.2017.20 28546564 PMC5454438

[7] Baumgart DC Carding SR. Inflammatory bowel disease: cause and immunobiology. Lancet (2007) 369(9573):1627–40. 10.1016/S0140-6736(07)60750-8 17499605

[8] Braus NA Elliott DE. Advances in the pathogenesis and treatment of IBD. Clin Immunol (2009) 132(1):1–9. 10.1016/j.clim.2009.02.006 19321388 PMC2693446

[9] Rutgeerts P Vermeire S Van AG. Biological therapies for inflammatory bowel diseases. Gastroenterology (2009) 136(4):1182–97. 10.1053/j.gastro.2009.02.001 19249397

[10] Ananthakrishnan AN. Epidemiology and risk factors for IBD. Nat Rev Gastroenterol Hepatol (2015) 12(4):205–17. 10.1038/nrgastro.2015.34 25732745

[11] Cao SS Zimmermann EM Chuang BM Song B Nwokoye A Wilkinson JE The unfolded protein response and chemical chaperones reduce protein misfolding and colitis in mice. Gastroenterology (2013) 144(5):989–1000.e6. 10.1053/j.gastro.2013.01.023 23336977 PMC3751190

[12] Hetz C. The unfolded protein response: controlling cell fate decisions under ER stress and beyond. Nat Rev Mol Cell Biol (2012) 13(2):89–102. 10.1038/nrm3270 22251901

[13] Marciniak SJ Ron D. Endoplasmic reticulum stress signaling in disease. Physiol Rev (2006) 86(4):1133–49. 10.1152/physrev.00015.2006 17015486

[14] Kaser A Blumberg RS. Endoplasmic reticulum stress in the intestinal epithelium and inflammatory bowel disease. Semin Immunol (2009) 21(3):156–63. 10.1016/j.smim.2009.01.001 19237300 PMC4736746

[15] Cao SS. Endoplasmic reticulum stress and unfolded protein response in inflammatory bowel disease. Inflamm Bowel Dis (2015) 21(3):636–44. 10.1097/MIB.0000000000000238 25581827

[16] Cao SS Kaufman RJ. Endoplasmic reticulum stress and oxidative stress in cell fate decision and human disease. Antioxid Redox Signal (2014) 21(3):396–413. 10.1089/ars.2014.5851 24702237 PMC4076992

[17] Walter P Ron D. The unfolded protein response: from stress pathway to homeostatic regulation. Science (2011) 334(6059):1081–6. 10.1126/science.1209038 22116877

[18] Cao SS Kaufman RJ Unfolded protein response. Curr Biol (2012) 22(16):319–25. 10.1016/j.cub.2012.07.004 22917505

[19] Tabas I Ron D. Integrating the mechanisms of apoptosis induced by endoplasmic reticulum stress. Nat Cell Biol (2011) 13(3):184–90. 10.1038/ncb0311-184 21364565 PMC3107571

[20] Cox JS Shamu CE Walter P. Transcriptional induction of genes encoding endoplasmic reticulum resident proteins requires a transmembrane protein kinase. Cell (1993) 73(6):1197–206. 10.1016/0092-8674(93)90648-A 8513503

[21] Shi Y Vattem KM Sood R An J Liang J Stramm L Identification and characterization of pancreatic eukaryotic initiation factor 2 α-subunit kinase, PEK, involved in translational control. J Biol Chem (1998) 252(9):2978–83. 10.1128/mcb.18.12.7499 PMC109330 9819435

[22] Haze K Yoshida H Yanagi H Yura T Mori K. Mammalian transcription factor ATF6 is synthesized as a transmembrane protein and activated by proteolysis in response to endoplasmic reticulum stress. Mol Biol Cell (1999) 10(11):3787–99. 10.1091/mbc.10.11.3787 10564271 PMC25679

[23] Bertolotti A Zhang Y Hendershot LM Harding HP Ron D. Dynamic interaction of BiP and ER stress transducers in the unfolded-protein response. Nat Cell Biol (2000) 2(6):326–32. 10.1038/35014100 10854322

[24] Shen J Chen X Hendershot L Prywes R. ER stress regulation of ATF6 localization by dissociation of BiP/GRP78 binding and unmasking of Golgi localization signals. Dev Cell (2002) 3(1):99–111. 10.1016/S1534-5807(02)00203-4 12110171

[25] Kimata Y Kimata YI Shimizu Y Abe H Farcasanu IC Takeuchi M Genetic evidence for a role of BiP/Kar2 that regulates Ire1 in response to accumulation of unfolded proteins. Mol Biol Cell (2003) 14(6):2559–69. 10.1091/mbc.E02-11-0708 12808051 PMC194903

[26] Ron D Walter P. Signal integration in the endoplasmic reticulum unfolded protein response. Nat Rev Mol Cell Biol (2007) 8(7):519–29. 10.1038/nrm2199 17565364

[27] Chen X Shen J Prywes R. The luminal domain of ATF6 senses endoplasmic reticulum (ER) stress and causes translocation of ATF6 from the ER to the Golgi. J Biol Chem (2002) 277(15):13045–52. 10.1074/jbc.M110636200 11821395

[28] Mori K Ma W Gething MJ Sambrook J. A transmembrane protein with a cdc2+/CDC28-related kinase activity is required for signaling from the ER to the nucleus. Cell (1993) 74(4):743–56. 10.1016/0092-8674(93)90521-Q 8358794

[29] Wang XZ Harding HP Zhang Y Jolicoeur EM Kuroda M Ron D. Cloning of mammalian Ire1 reveals diversity in the ER stress responses. EMBO J (1998) 17(19):5708–17. 10.1093/emboj/17.19.5708 9755171 PMC1170899

[30] Bertolotti A Wang X Novoa I Jungreis R Schlessinger K Cho JH Increased sensitivity to dextran sodium sulfate colitis in IRE1β-deficient mice. J Clin Invest (2001) 107(5):585–93. 10.1172/JCI11476 11238559 PMC199427

[31] Gardner BM Pincus D Gotthardt K Gallagher CM Walter P. Endoplasmic reticulum stress sensing in the unfolded protein response. Cold Spring Harb Perspect Biol (2013) 5(3):a013169. 10.1101/cshperspect.a013169 23388626 PMC3578356

[32] Gardner BM Walter P. Unfolded proteins are Ire1-activating ligands that directly induce the unfolded protein response. Science (2011) 333(6051):1891–4. 10.1126/science.1209126 21852455 PMC3202989

[33] Marcella C Huiqing Z Fumihiko U Till JH Hubbard SR Harding HP IRE1 couples endoplasmic reticulum load to secretory capacity by processing the *XBP-1* mRNA. Nature (2002) 415(6867):92–6. 10.1038/415092a 11780124

[34] Yoshida H Matsui T Yamamoto A Okada T Mori K. XBP1 mRNA is induced by ATF6 and spliced by IRE1 in response to ER stress to produce a highly active transcription factor. Cell (2001) 107(7):881–91. 10.1016/S0092-8674(01)00611-0 11779464

[35] Lee K Tirasophon W Shen X Michalak M Prywes R Okada T IRE1-mediated unconventional mRNA splicing and S2P-mediated ATF6 cleavage merge to regulate XBP1 in signaling the unfolded protein response. Genes Dev (2002) 16(16):452–66. 10.1101/gad.964702 11850408 PMC155339

[36] Lee AH Scapa EF Cohen DE Glimcher LH. Regulation of hepatic lipogenesis by the transcription factor XBP1. Science (2008) 320(5882):1492–6. 10.1126/science.1158042 18556558 PMC3620093

[37] Martinon F Chen X Lee AH Glimcher LH Toll-like receptor activation of XBP1 regulates innate immune responses in macrophages. Nat Immunol (2010) 11(5):411–8. 10.1038/ni.1857 20351694 PMC3113706

[38] Chen X Iliopoulos D Zhang Q Tang Q Greenblatt MB Hatziapostolou M XBP1 promotes triple-negative breast cancer by controlling the HIF1α pathway. Nature (2014) 508(7494):103–7. 10.1038/nature13119 24670641 PMC4105133

[39] Acosta-Alvear D Zhou Y Blais A Tsikitis M Lents NH Arias C XBP1 controls diverse cell type- and condition-specific transcriptional regulatory networks. Mol Cell (2007) 27(1):53–66. 10.1016/j.molcel.2007.06.011 17612490

[40] Wang ZV Deng Y Gao N Pedrozo Z Li DL Morales CR Spliced X-box binding protein 1 couples the unfolded protein response to hexosamine biosynthetic pathway. Cell (2014) 156(6):1179–92. 10.1016/j.cell.2014.01.014 24630721 PMC3959665

[41] Lee AH Iwakoshi NN Anderson KC Glimcher LH. Proteasome inhibitors disrupt the unfolded protein response in myeloma cells. Proc Natl Acad Sci U S A (2003) 100(17):9946–51. 10.1073/pnas.1334037100 12902539 PMC187896

[42] Yoshida H Oku M Suzuki M Mori K. pXBP1(U) encoded in XBP1 pre-mRNA negatively regulates unfolded protein response activator pXBP1(S) in mammalian ER stress response. J Cell Biol (2006) 172(4):565–75. 10.1083/jcb.200508145 16461360 PMC2063676

[43] Lee AH Iwakoshi NN Glimcher LH. XBP-1 regulates a subset of endoplasmic reticulum resident chaperone genes in the unfolded protein response. Mol Cell Biol (2003) 23(21):7448–59. 10.1128/MCB.23.21.7448-7459.2003 14559994 PMC207643

[44] Hollien J Weissman JS. Decay of endoplasmic reticulum-localized mRNAs during the unfolded protein response. Science (2006) 313(5783):104–7. 10.1126/science.1129631 16825573

[45] Hollien J Lin JH Li H Stevens N Walter P Weissman JS. Regulated Ire1-dependent decay of messenger RNAs in mammalian cells. J Cell Biol (2009) 186(3):323–31. 10.1083/jcb.200903014 19651891 PMC2728407

[46] Bommiasamy H Back SH Fagone P Lee K Meshinchi S Vink E ATF6alpha induces XBP1-independent expansion of the endoplasmic reticulum. J Cell Sci (2009) 122(Pt 10):1626–36. 10.1242/jcs.045625 19420237 PMC2680102

[47] Nguyên DT Kebache S Fazel A Wong HN Jenna S Emadali A Nck-dependent activation of extracellular signal-regulated kinase-1 and regulation of cell survival during endoplasmic reticulum stress. Mol Biol Cell (2004) 15(9):4248–60. 10.1091/mbc.E03-11-0851 15201339 PMC515356

[48] Hetz C Bernasconi P Fisher J Lee AH Bassik MC Antonsson B Proapoptotic BAX and BAK modulate the unfolded protein response by a direct interaction with IRE1α. Science (2006) 312(5773):572–6. 10.1126/science.1123480 16645094

[49] Upton JP Wang L Han D Wang ES Huskey NE Lim L IRE1α cleaves select microRNAs during ER stress to derepress translation of proapoptotic caspase-2. Science (2012) 338(6108):818–22. 10.1126/science.1226191 23042294 PMC3742121

[50] Hassler J Cao SS Kaufman RJ. IRE1, a double-edged sword in pre-miRNA slicing and cell death. Dev Cell (2012) 23(5):921–3. 10.1016/j.devcel.2012.10.025 23153490 PMC3684431

[51] Lerner AG Upton JP Praveen PV Ghosh R Nakagawa Y Igbaria A IRE1alpha induces thioredoxin-interacting protein to activate the NLRP3 inflammasome and promote programmed cell death under irremediable ER stress. Cell Metab (2012) 16(2):250–64. 10.1016/j.cmet.2012.07.007 22883233 PMC4014071

[52] Nakamura D Tsuru A Ikegami K Imagawa Y Fujimoto N Kohno K. Mammalian ER stress sensor IRE1β specifically down-regulates the synthesis of secretory pathway proteins. FEBS Lett (2011) 585(1):133–8. 10.1016/j.febslet.2010.12.002 21146530

[53] Harding HP Zhang Y Ron D. Protein translation and folding are coupled by an endoplasmic-reticulum-resident kinase. Nature (1999) 397(6716):271–4. 10.1038/16729 9930704

[54] McGuckin MA Eri RD Das I Lourie R Florin TH. ER stress and the unfolded protein response in intestinal inflammation. Am J Physiol Gastrointest Liver Physiol (2010) 298(6):G820–32. 10.1152/ajpgi.00063.2010 20338921

[55] Kaser A Blumberg RS. Endoplasmic reticulum stress and intestinal inflammation. Mucosal Immunol (2010) 3(1):11–6. 10.1038/mi.2009.122 19865077 PMC4592136

[56] Harding HP Zhang Y Bertolotti A Zeng H Ron D. *Perk* is essential for translational regulation and cell survival during the unfolded protein response. Mol Cell (2000) 5(5):897–904. 10.1016/S1097-2765(00)80330-5 10882126

[57] Harding HP Zhang Y Zeng H Novoa I Lu PD Calfon M An integrated stress response regulates amino acid metabolism and resistance to oxidative stress. Mol Cell (2003) 11(3):619–33. 10.1016/S1097-2765(03)00105-9 12667446

[58] Vattem KM Wek RC. Reinitiation involving upstream ORFs regulates ATF4 mRNA translation in mammalian cells. Proc Natl Acad Sci U S A (2004) 101(31):11269–74. 10.1073/pnas.0400541101 15277680 PMC509193

[59] Sano R Reed JC. ER stress-induced cell death mechanisms. Biochim Biophys Acta (2013) 1833(12):3460–70. 10.1016/j.bbamcr.2013.06.028 23850759 PMC3834229

[60] Szegezdi E Logue SE Gorman AM Samali A Mediators of endoplasmic reticulum stress-induced apoptosis. EMBO Rep (2006) 7(9):880–5. 10.1038/sj.embor.7400779 16953201 PMC1559676

[61] Urra H Dufey E Lisbona F Rojas-Rivera D Hetz C. When ER stress reaches a dead end. Biochim Biophys Acta (2013) 1833(12):3507–17. 10.1016/j.bbamcr.2013.07.024 23988738

[62] Lu M Lawrence DA Marsters S Acostaalvear D Kimmig P Mendez AS Opposing unfolded-protein-response signals converge on death receptor 5 to control apoptosis. Science (2014) 345(6192):98–101. 10.1126/science.1254312 24994655 PMC4284148

[63] Han J Back SH Hur J Lin YH Gildersleeve R Shan J ER-stress-induced transcriptional regulation increases protein synthesis leading to cell death. Nat Cell Biol (2013) 15(5):481–90. 10.1038/ncb2738 23624402 PMC3692270

[64] Zhang K Kaufman RJ. From endoplasmic-reticulum stress to the inflammatory response. Nature (2008) 454(7203):455–62. 10.1038/nature07203 18650916 PMC2727659

[65] Mathers J Fraser JA McMahon M Saunders RD Hayes JD McLellan LI Antioxidant and cytoprotective responses to redox stress. Biochem Soc Symp (2004) (71):157–76. 10.1042/bss0710157 15777020

[66] Zhang DD. Mechanistic studies of the Nrf2-Keap1 signaling pathway. Drug Metab Rev (2006) 38(4):769–89. 10.1080/03602530600971974 17145701

[67] Nakamura T Furuhashi M Li P Cao HM Tuncman G Sonenberg N Double-stranded RNA-dependent protein kinase links pathogen sensing with stress and metabolic homeostasis. Cell (2010) 140(3):338–48. 10.1016/j.cell.2010.01.001 20144759 PMC2820414

[68] Murguia JR Serrano R. New functions of protein kinase Gcn2 in yeast and mammals. IUBMB Life (2012) 64(12):971–4. 10.1002/iub.1090 23129244

[69] Wek RC Jiang HY Anthony TG. Coping with stress: eIF2 kinases and translational control. Biochem Soc Trans (2006) 34(1):7–11. 10.1042/BST0340007 16246168

[70] Joshi M Kulkarni A Pal JK Small molecule modulators of eukaryotic initiation factor 2alpha kinases, the key regulators of protein synthesis. Biochimie (2013) 95(11):1980–90. 10.1016/j.biochi.2013.07.030 23939221

[71] Donnelly N Gorman AM Gupta S Samali A The eIF2alpha kinases: their structures and functions. Cell Mol Life Sci (2013) 70(19):3493–511. 10.1007/s00018-012-1252-6 23354059 PMC11113696

[72] Connor JH Weiser DC Li S Hallenbeck JM Shenolikar S. Growth arrest and DNA damage-inducible protein GADD34 assembles a novel signaling complex containing protein phosphatase 1 and inhibitor 1. Mol Cell Biol (2001) 21(20):6841–50. 10.1128/MCB.21.20.6841-6850.2001 11564868 PMC99861

[73] Brush MH Weiser DC Shenolikar S Growth arrest and DNA damage-inducible protein GADD34 targets protein phosphatase 1α to the endoplasmic reticulum and promotes dephosphorylation of the α subunit of eukaryotic translation initiation factor 2. Mol Cell Biol (2003) 23(4):1292–303. 10.1128/MCB.23.4.1292-1303.2003 12556489 PMC141149

[74] Chambers JE Dalton LE Clarke HJ Malzer E Dominicus CS Patel V Actin dynamics tune the integrated stress response by regulating eukaryotic initiation factor 2alpha dephosphorylation. Elife (2015) 4(4):822–3. 10.7554/eLife.04872 PMC4394351 25774599

[75] Thuerauf DJ Morrison L Glembotski CC Opposing roles for ATF6α and ATF6β in endoplasmic reticulum stress response gene induction. J Biol Chem (2004) 279(279):21078–84. 10.1074/jbc.M400713200 14973138

[76] Yoshida H Haze K Yanagi H Yura T Mori K. Identification of the cis-acting endoplasmic reticulum stress response element responsible for transcriptional induction of mammalian glucose-regulated proteins. Involvement of basic leucine zipper transcription factors. J Biol Chem (1998) 273(50):33741–9. 10.1074/jbc.273.50.33741 9837962

[77] Kokame K Kato H Miyata T. Identification of ERSE-II, a new cis-acting element responsible for the ATF6-dependent mammalian unfolded protein response. J Biol Chem (2001) 276(12):9199–205. 10.1074/jbc.M010486200 11112790

[78] Yamamoto K Sato T Matsui T Sato M Okada T Yoshida H Transcriptional induction of mammalian ER quality control proteins is mediated by single or combined action of ATF6α and XBP1. Dev Cell (2007) 13(3):365–76. 10.1016/j.devcel.2007.07.018 17765680

[79] Namba T Ishihara T Tanaka K Hoshino T Mizushima T. Transcriptional activation of ATF6 by endoplasmic reticulum stressors. Biochem Biophys Res Commun (2007) 355(2):543–8. 10.1016/j.bbrc.2007.02.004 17307147

[80] Kondo S Saito A Asada R Kanemoto S Imaizumi K. Physiological unfolded protein response regulated by OASIS family members, transmembrane bZIP transcription factors. IUBMB Life (2011) 63(4):233–9. 10.1002/iub.433 21438114

[81] Asada R Kanemoto S Kondo S Saito A Imaizumi K. The signalling from endoplasmic reticulum-resident bZIP transcription factors involved in diverse cellular physiology. J Biochem (2011) 149(5):507–18. 10.1093/jb/mvr041 21454302

[82] Kondo S Murakami T Tatsumi K Ogata M Kanemoto S Otori K OASIS, a CREB/ATF-family member, modulates UPR signalling in astrocytes. Nat Cell Biol (2005) 7(2):186–94. 10.1038/ncb1213 15665855

[83] Zhang K Shen X Wu J Sakaki K Saunders T Rutkowski DT Endoplasmic reticulum stress activates cleavage of CREBH to induce a systemic inflammatory response. Cell (2006) 124(3):587–99. 10.1016/j.cell.2005.11.040 16469704

[84] Kondo S Saito A Hino S Murakami T Ogata M Kanemoto S BBF2H7, a novel transmembrane bZIP transcription factor, is a new type of endoplasmic reticulum stress transducer. Mol Cell Biol (2007) 27(5):1716–29. 10.1128/MCB.01552-06 17178827 PMC1820470

[85] Nagamori I Yabuta N Fujii T Tanaka H Yomogida K Nishimune Y Tisp40, a spermatid specific bZip transcription factor, functions by binding to the unfolded protein response element via the Rip pathway. Genes Cells (2005) 10(6):575–94. 10.1111/j.1365-2443.2005.00860.x 15938716

[86] Liang G Audas TE Li Y Cockram GP Dean JD Martyn AC Luman/CREB3 induces transcription of the endoplasmic reticulum (ER) stress response protein Herp through an ER stress response element. Mol Cell Biol (2006) 26(21):7999–8010. 10.1128/MCB.01046-06 16940180 PMC1636730

[87] DenBoer LM Hardy-Smith PW Hogan MR Cockram GP Audas TE Lu R. Luman is capable of binding and activating transcription from the unfolded protein response element. Biochem Biophys Res Commun (2005) 331(1):113–9. 10.1016/j.bbrc.2005.03.141 15845366

[88] Kondo S Hino SI Saito A Kanemoto S Kawasaki N Asada R Activation of OASIS family, ER stress transducers, is dependent on its stabilization. Cell Death Differ (2012) 19(12):1939–49. 10.1038/cdd.2012.77 22705851 PMC3504707

[89] Bailey D O’Hare P. Transmembrane bZIP transcription factors in ER stress signaling and the unfolded protein response. Antioxid Redox Signal (2007) 9(12):2305–22. 10.1089/ars.2007.1796 17887918

[90] Mcguckin MA Eri RD Das I Lourie R Florin TH. Intestinal secretory cell ER stress and inflammation. Biochem Soc Trans (2011) 39(4):1081–5. 10.1042/BST0391081 21787352

[91] Harrison E Lal S Mclaughlin JT. Enteroendocrine cells in gastrointestinal pathophysiology. Curr Opin Pharmacol (2013) 13(6):941–5. 10.1016/j.coph.2013.09.012 24206752

[92] Vaishnava S Behrendt CL Ismail AS Eckmann L Hooper LV. Paneth cells directly sense gut commensals and maintain homeostasis at the intestinal host-microbial interface. Proc Natl Acad Sci U S A (2008) 105(52):20858–63. 10.1073/pnas.0808723105 19075245 PMC2603261

[93] Dohrman A Miyata S Gallup M Li JD Chapelin C Coste A Mucin gene (MUC 2 and MUC 5AC) upregulation by Gram-positive and Gram-negative bacteria. Biochim Biophys Acta (1998) 1406(3):251–9. 10.1016/S0925-4439(98)00010-6 9630659

[94] O’Neil DA Porter EM Elewaut D Anderson GM Eckmann L Ganz T Expression and regulation of the human beta-defensins hBD-1 and hBD-2 in intestinal epithelium. J Immunol (1999) 163(12):6718–24. 10586069

[95] Schröder M Kaufman RJ The mammalian unfolded protein response. Biochemistry (2005) 74(74):739–89. 10.1146/annurev.biochem.73.011303.074134 15952902

[96] Wehkamp J Salzman NH Porter E Nuding S Weichenthal M Petras RE Reduced Paneth cell α-defensins in ileal Crohn’s disease. Proc Natl Acad Sci U S A (2005) 102(50):18129–34. 10.1073/pnas.0505256102 16330776 PMC1306791

[97] Kaser A Lee A Franke A Glickman J Zeissig S Tilg H XBP1 links ER stress to intestinal inflammation and confers genetic risk for human inflammatory bowel disease. Cell (2008) 134(5):743–56. 10.1016/j.cell.2008.07.021 18775308 PMC2586148

[98] Heazlewood CK Cook MC Eri R Price GR Tauro SB Taupin D Aberrant mucin assembly in mice causes endoplasmic reticulum stress and spontaneous inflammation resembling ulcerative colitis. PLoS Med (2008) 5(3):e54. 10.1371/journal.pmed.0050054 18318598 PMC2270292

[99] Shkoda A Ruiz PA Daniel H Kim SC Rogler G Sartor RB Interleukin-10 blocked endoplasmic reticulum stress in intestinal epithelial cells: impact on chronic inflammation. Gastroenterology (2007) 132(1):190–207. 10.1053/j.gastro.2006.10.030 17241871

[100] Tsuru A Fujimoto N Takahashi S Saito M Nakamura D Iwano M Negative feedback by IRE1β optimizes mucin production in goblet cells. Proc Natl Acad Sci U S A (2013) 110(8):2864–9. 10.1073/pnas.1212484110 23386727 PMC3581977

[101] Zhang HS Chen Y Fan L Xi QL Wu GH Li XX The endoplasmic reticulum stress sensor IRE1α in intestinal epithelial cells is essential for protecting against colitis. J Biol Chem (2015) 290(24):15327–36. 10.1074/jbc.M114.633560 25925952 PMC4463471

[102] Hampe J Schreiber S Shaw SH Lau KF Bridger S Macpherson AJ A genomewide analysis provides evidence for novel linkages in inflammatory bowel disease in a large European cohort. Am J Hum Genet (1999) 64(3):808–16. 10.1086/302294 10053016 PMC1377799

[103] Vermeire S Rutgeerts P Van Steen K Joossens S Claessens G Pierik M Genome wide scan in a Flemish inflammatory bowel disease population: support for the IBD4 locus, population heterogeneity, and epistasis. Gut (2004) 53(7):980–6. 10.1136/gut.2003.034033 15194648 PMC1774099

[104] Oyadomari S Mori M. Roles of CHOP/GADD153 in endoplasmic reticulum stress. Cell Death Differ (2004) 11(4):381–9. 10.1038/sj.cdd.4401373 14685163

[105] Park SH Choi HJ Yang H Do KH Kim J Lee DW Endoplasmic reticulum stress-activated C/EBP homologous protein enhances nuclear factor-κB signals via repression of peroxisome proliferator-activated receptor γ. J Biol Chem (2010) 285(46):35330–9. 10.1074/jbc.M110.136259 20829347 PMC2975157

[106] Namba T Tanaka K Ito Y Ishihara T Hoshino T Gotoh T Positive role of CCAAT/enhancer-binding protein homologous protein, a transcription factor involved in the endoplasmic reticulum stress response in the development of colitis. Am J Pathol (2009) 174(5):1786–98. 10.2353/ajpath.2009.080864 19359519 PMC2671267

[107] Abraham C Cho JH. IL-23 and autoimmunity: new insights into the pathogenesis of inflammatory bowel disease. Annu Rev Med (2009) 60:97–110. 10.1146/annurev.med.60.051407.123757 18976050

[108] Duerr RH Taylor KD Brant SR Rioux JD Silverberg MS Daly MJ A genome-wide association study identifies IL23R as an inflammatory bowel disease gene. Science (2006) 314(5804):1461–3. 10.1126/science.1135245 17068223 PMC4410764

[109] Hölttä V Klemetti P Sipponen T Westerholm-Ormio M Kociubinski G Salo H IL-23/IL-17 immunity as a hallmark of Crohn’s disease. Inflamm Bowel Dis (2008) 14(9):1175–84. 10.1002/ibd.20475 18512248

[110] Geremia A Jewell DP. The IL-23/IL-17 pathway in inflammatory bowel disease. Expert Rev Gastroenterol Hepatol (2012) 6(2):223–37. 10.1586/egh.11.107 22375527

[111] Sarra M Pallone F Macdonald TT Monteleone G. IL-23/IL-17 axis in IBD. Inflamm Bowel Dis (2010) 16(10):1808–13. 10.1002/ibd.21248 20222127

[112] Hue S Ahern P Buonocore S Kullberg MC Cua DJ Mckenzie BS Interleukin-23 drives innate and T cell-mediated intestinal inflammation. J Exp Med (2006) 203(11):2473–83. 10.1084/jem.20061099 17030949 PMC2118132

[113] Izcue A Hue S Buonocore S Arancibiacárcamo CV Ahern PP Iwakura Y Interleukin-23 restrains regulatory T cell activity to drive T cell-dependent colitis. Immunity (2008) 28(4):559–70. 10.1016/j.immuni.2008.02.019 18400195 PMC2292821

[114] Maloy KJ Powrie F. Intestinal homeostasis and its breakdown in inflammatory bowel disease. Nature (2011) 474(7351):298–306. 10.1038/nature10208 21677746

[115] Buonocore S Ahern PP Uhlig HH Ivanov II Dan RL Maloy KJ Innate lymphoid cells drive IL-23 dependent innate intestinal pathology. Nature (2010) 464(7293):1371–5. 10.1038/nature08949 20393462 PMC3796764

[116] Verstockt B Van AG Vermeire S Ferrante M Biological therapy targeting the IL-23/IL-17 axis in inflammatory bowel disease. Expert Opin Biol Ther (2017) 17(1):31–47. 10.1080/14712598.2017.1258399 27817215

[117] Goodall JC Wu C Zhang Y McNeill L Ellis L Saudek V Endoplasmic reticulum stress-induced transcription factor, CHOP, is crucial for dendritic cell IL-23 expression. Proc Natl Acad Sci U S A (2010) 107(41):17698–703. 10.1073/pnas.1011736107 20876114 PMC2955096

[118] Dubois M ATF6α optimizes long-term endoplasmic reticulum function to protect cells from chronic stress. Dev Cell (2007) 13(3):351–64. 10.1016/j.devcel.2007.07.005 17765679

[119] Hotamisligil GS. Endoplasmic reticulum stress and the inflammatory basis of metabolic disease. Cell (2010) 140(6):900–17. 10.1016/j.cell.2010.02.034 20303879 PMC2887297

[120] Kroemer G Mariño G Levine B. Autophagy and the integrated stress response. Mol Cell (2010) 40(2):280–93. 10.1016/j.molcel.2010.09.023 20965422 PMC3127250

[121] Brandl K Rutschmann S Li X Du X Xiao N Schnabl B Enhanced sensitivity to DSS colitis caused by a hypomorphic Mbtps1 mutation disrupting the ATF6-driven unfolded protein response. Proc Natl Acad Sci U S A (2009) 106(9):3300–5. 10.1073/pnas.0813036106 19202076 PMC2651297

[122] Ye J Rawson RB Komuro R Chen X Dave UP Prywes R ER stress induces cleavage of membrane-bound ATF6 by the same proteases that process SREBPs. Mol Cell (2000) 6(6):1355–64. 10.1016/S1097-2765(00)00133-7 11163209

[123] Asada R Saito A Kawasaki N Kanemoto S Iwamoto H Oki M Endoplasmic reticulum stress transducer OASIS promotes terminal differentiation of goblet cells in large intestine. J Biol Chem (2012) 287(11):8144–53. 10.1074/jbc.M111.332593 22262831 PMC3318704

[124] Hino K Saito A Asada R Kanemoto S Imaizumi K. Increased susceptibility to dextran sulfate sodium-induced colitis in the endoplasmic reticulum stress transducer OASIS deficient mice. PLoS One (2014) 9(2):e88048. 10.1371/journal.pone.0088048 24498426 PMC3912207

[125] Fang Z Edwards R Dizon D Afrasiabi K Mastroianni JR Geyfman M Disruption of Paneth and goblet cell homeostasis and increased endoplasmic reticulum stress in *Agr2-/-* mice. Dev Biol (2010) 338(2):270–9. 10.1016/j.ydbio.2009.12.008 20025862 PMC2937056

[126] Wang Z Hao Y Lowe AW. The adenocarcinoma-associated antigen, AGR2, promotes tumor growth, cell migration, and cellular transformation. Cancer Res (2008) 68(2):492–7. 10.1158/0008-5472.CAN-07-2930 18199544

[127] Komiya T Tanigawa Y Hirohashi S. Cloning of the gene gob-4, which is expressed in intestinal goblet cells in mice. Biochim Biophys Acta (1999) 1444(3):434–8. 10.1016/S0167-4781(99)00010-X 10095068

[128] Ellgaard L Ruddock LW. The human protein disulphide isomerase family: substrate interactions and functional properties. EMBO Rep (2005) 6(1):28–32. 10.1038/sj.embor.7400311 15643448 PMC1299221

[129] Zheng W Rosenstiel P Huse K Sina C Valentonyte R Mah N Evaluation of AGR2 and AGR3 as candidate genes for inflammatory bowel disease. Genes Immun (2006) 7(1):11–8. 10.1038/sj.gene.6364263 16222343

[130] Johansson MEV Larsson JMH Hansson GC. The two mucus layers of colon are organized by the MUC2 mucin, whereas the outer layer is a legislator of host-microbial interactions. Proc Natl Acad Sci U S A (2011) 108(Suppl 1):4659–65. 10.1073/pnas.1006451107 20615996 PMC3063600

[131] Hansson GC. Role of mucus layers in gut infection and inflammation. Curr Opin Microbiol (2012) 15(1):57–62. 10.1016/j.mib.2011.11.002 22177113 PMC3716454

[132] Kim YS Ho SB. Intestinal goblet cells and mucins in health and disease: recent insights and progress. Curr Gastroenterol Rep (2010) 12(5):319–30. 10.1007/s11894-010-0131-2 20703838 PMC2933006

[133] Hanski C Born M Foss HD Marowski B Mansmann U Arastéh K Defective post-transcriptional processing of MUC2 mucin in ulcerative colitis and in Crohn’s disease increases detectability of the MUC2 protein core. J Pathol (1999) 188(3):304–11. 10.1002/(SICI)1096-9896(199907)188:3<304::AID-PATH375>3.0.CO;2-A 10419600

[134] Xue X Piao JH Nakajima A Sakon-Komazawa S Kojima Y Mori K Tumor necrosis factor alpha (TNFα) induces the unfolded protein response (UPR) in a reactive oxygen species (ROS)-dependent fashion, and the UPR counteracts ROS accumulation by TNFα. J Biol Chem (2005) 280(40):33917–25. 10.1074/jbc.M505818200 16107336

[135] Tashiro E Hironiwa N Kitagawa M Futamura Y Suzuki S Nishio M Trierixin, a novel inhibitor of ER stress-induced XBP1 activation from *Streptomyces* sp. 1. Taxonomy, fermentation, isolation and biological activities. J Antibiot (2008) 60(3):547–53. 10.1038/ja.2007.69 17917237

[136] Paton AW Potjanee S Talbot UM Hui W Paton JC. A new family of potent AB(5) cytotoxins produced by Shiga toxigenic *Escherichia coli*. J Exp Med (2004) 200(1):35–46. 10.1084/jem.20040392 15226357 PMC2213318

[137] Kühn R Löhler J Rennick D Rajewsky K Müller W Interleukin-10-deficient mice develop chronic enterocolitis. Cell (1993) 75(2):263–74. 10.1016/0092-8674(93)80068-P 8402911

[138] Xavier RJ Podolsky DK. Unravelling the pathogenesis of inflammatory bowel disease. Nature (2007) 448(7152):427–34. 10.1038/nature06005 17653185

[139] Strober W Fuss IJ Blumberg RS. The immunology of mucosal models of inflammation. Immunology (2002) 20(20):495–549. 10.1146/annurev.immunol.20.100301.064816 11861611

[140] Paton AW Beddoe T Thorpe CM Whisstock JC Wilce MC Rossjohn J AB5 subtilase cytotoxin inactivates the endoplasmic reticulum chaperone BiP. Nature (2006) 443(7111):548–52. 10.1038/nature05124 17024087

[141] Abraham C Cho JH Inflammatory bowel disease. N Engl J Med (1991) 361(21):2066–78. 10.1056/NEJMra0804647 PMC3491806 19923578

[142] Powers ET Morimoto RI Dillin A Kelly JW Balch WE. Biological and chemical approaches to diseases of proteostasis deficiency. Annu Rev Biochem (2009) 78:959–91. 10.1146/annurev.biochem.052308.114844 19298183

[143] Xiao C Giacca A Lewis GF. Sodium phenylbutyrate, a drug with known capacity to reduce endoplasmic reticulum stress, partially alleviates lipid-induced insulin resistance and beta-cell dysfunction in humans. Diabetes (2011) 60(3):918–24. 10.2337/db10-1433 21270237 PMC3046853

[144] Kars M Yang L Gregor MF Mohammed BS Pietka TA Finck BN Tauroursodeoxycholic acid may improve liver and muscle but not adipose tissue insulin sensitivity in obese men and women. Diabetes (2010) 59(8):1899–905. 10.2337/db10-0308 20522594 PMC2911053

[145] Ozcan U Yilmaz E Ozcan L Furuhashi M Vaillancourt E Smith RO Chemical chaperones reduce ER stress and restore glucose homeostasis in a mouse model of type 2 diabetes. Science (2006) 313(5790):1137–40. 10.1126/science.1128294 16931765 PMC4741373

[146] Berger E Haller D Structure–function analysis of the tertiary bile acid TUDCA for the resolution of endoplasmic reticulum stress in intestinal epithelial cells. Biochem Biophys Res Commun (2011) 409(4):610–5. 10.1016/j.bbrc.2011.05.043 21605547

[147] Tabata Y Takano K Ito T Iinuma M Yoshimoto T Miura H Vaticanol B, a resveratrol tetramer, regulates endoplasmic reticulum stress and inflammation. Am J Physiol Cell Physiol (2007) 293(1):411–8. 10.1152/ajpcell.00095.2007 17475668

[148] Greaves MW Tan KT. Chronic urticaria: recent advances. Clin Rev Allergy Immunol (2007) 33(1):134–43. 10.1007/s12016-007-0038-3 18094952

[149] Koh SJ Kim JW Kim BG Lee KL Chun J Kim JS Fexofenadine regulates nuclear factor-κB signaling and endoplasmic reticulum stress in intestinal epithelial cells and ameliorates acute and chronic colitis in mice. J Pharmacol Exp Ther (2014) 352(3):455–61. 10.1124/jpet.114.217844 25538104

[150] Okazaki T Nishio A Takeo M Sakaguchi Y Fukui T Uchida K Inhibition of the dephosphorylation of eukaryotic initiation factor 2α ameliorates murine experimental colitis. Digestion (2014) 90(3):167–78. 10.1159/000366414 25339182

[151] Boyce M Yuan J A selective inhibitor of eIF2α dephosphorylation protects cells from ER stress. Science (2005) 307(5711):935–9. 10.1126/science.1101902 15705855

[152] Crespo I San-Miguel B Prause C Marroni N Cuevas MJ González-Gallego J Glutamine treatment attenuates endoplasmic reticulum stress and apoptosis in TNBS-induced colitis. PLoS One (2012) 7(11):e50407. 10.1371/journal.pone.0050407 23209735 PMC3508929

[153] Wang B Wu Z Ji Y Sun K Dai Z Wu G. L-glutamine enhances tight junction integrity by activating CaMK kinase 2-AMP-activated protein kinase signaling in intestinal porcine epithelial cells. J Nutr (2016) 146(3):501–8. 10.3945/jn.115.224857 26865645

